# The Principle of Patients' Payments

**Published:** 1920-11-20

**Authors:** A. E. Cummins

**Affiliations:** St. Thomas's Hospital, Westminster Bridge, S.E. 1


### THE EDITOR'S LETTER-BOX.

**Correspondence on all subjects is invited, but we cannot in any way be responsible for the opinions expressed by our correspondents, who must give their name and address as a guarantee of good faith, but not necessarily for publication. Correspondents are reminded that brevity of style and conciseness of statement greatly facilitate early insertion.**

### The Principle of Patients' Payments.

*To the Editor of* THE HOSPITAL.

SIR,—My attention has been drawn to the excellent article on the principle of patients' payments in your issue of October 30, but may I point out that you are hardly fair in your concluding statement as to the attitude of the almoner in this matter? At this hospital my department has undertaken the whole responsibility, under the direction of the Governors, of arranging for and collecting the patients' contributions in addition to the already heavy work it had on hand; and I believe I am correct in stating that this is the case in many other hospitals.

If a flat rate is imposed it can doubtless be collected more or less mechanically. As the writer of your article observes, it must be left to experience to show whether the "best results" from the purely financial point of view will result from a flat rate or from assessment according to means.

No one who has the interests of our voluntary hospitals at heart can venture to question the very vital importance of finance, but as an almoner I hold that a flat rate is neither in the interests of the patient or the medical profession. Moreover, in this age of preventive medicine, it would surely tend to hinder many of the working classes from coming up to the hospital in the early stages of disease.

That, however, every patient should be urged to give in accordance with his or her means, is a fact that no one can dispute, and the Treasurer and Governors of this hospital have insisted on this principle for the last ten years, even when public opinion urged so strongly the open door for purely sentimental reasons.—Yours faithfully,

A. E. CUMMINS,

St. Thomas's Hospital, Westminster Bridge, S.E. 1.
November 8, 1920.

